# Role of ENaC in gender-associated differences in blood pressure

**DOI:** 10.22038/ijbms.2025.81832.17701

**Published:** 2025

**Authors:** Guo-feng Yu, Li-qin Yu, Qin-rui Lai, Wei Li

**Affiliations:** 1 Department of Clinical Laboratory, Children’s Hospital, Zhejiang University School of Medicine, Hangzhou, Zhejiang Province, China, 310052; 2 Qiaosi Branch, First People’s Hospital of Linping District, Hangzhou, Zhejiang Province, China; # These authors contributed equally to this work

**Keywords:** Castration, Epithelial sodium channel, Hypertension, Lysine-deficient protein – kinase, Sex differences

## Abstract

**Objective(s):**

Sexual dimorphism in blood pressure regulation has been extensively noted in humans, but the underlying mechanisms remain to be fully understood. Our research aims to investigate the possible correlation between gender-associated differences in blood pressure and renal sodium transport.

**Materials and Methods:**

We measured male and female mice’s blood pressure, urine, and plasma sodium concentration when fed a regular or high-Na^+^ diet. After that, their renal sodium transporters were assessed by western blot and immunofluorescence. For further investigation, male mice were castrated to observe the differences in blood pressure and renal sodium transporters compared to normal mice.

**Results:**

Male mice exhibited higher blood pressure and lower renal sodium excretion than female littermates. Furthermore, the blood pressure of male mice exhibited a more significant and rapid increase relative to female mice when the diet was switched from control sodium to high sodium. Western blot and immunofluorescent staining revealed that in male mice, the sodium transporters epithelial sodium channel (ENaC) and the upstream kinases SPAK (Ste20-related proline/alanine-rich kinase), OSR1 (oxidative stress response kinase 1), and WNK4 (Lysine-Deficient Protein Kinase 4) were elevated. Beyond that, male mice exhibited lowered blood pressure and reduced abundance of ENaC (α, β, and γ) after castration.

**Conclusion:**

ENaC plays a significant role in gender-associated differences in blood pressure and renal sodium reabsorption.

## Introduction

Men exhibit a higher prevalence and severity of hypertension than women (1-3), and this phenomenon was also found in animal models (4-7). However, the underlying mechanisms accounting for these disparities still need to be fully elucidated. It is well-known that renal sodium reabsorption plays a vital role in blood pressure regulation; nonetheless, whether the differences in renal sodium reabsorption account for the variation of blood pressure between the sexes needs further investigation.

The kidney maintains sodium homeostasis and blood pressure balance via renal sodium transporters. In particular, sodium transporters NCC and NKCC2 have been demonstrated to affect blood pressure regulation profoundly. Loss-of-function mutations in NCC and NKCC2, detected in patients, lead to salt wasting and hypotension (8-11). In contrast, hypertensive patients caused by WNK kinase mutation have shown an increased activation of NCC and sodium retention (12-14). The epithelial sodium channel (ENaC), composed of three homologous subunits (α, β, and γ), is indispensable in the process of renal Na^+^ transport (15, 16). A deficiency of ENaC results in salt wasting and hypotension (17-19). Furthermore, studies have shown that sex steroids can regulate renal ENaC mRNA expression in animals (20, 21). 

Research has demonstrated that ENaC, NCC, and NKCC2 are the downstream targets of SPAK, OSR1, and WNK kinases (22-25). WNK kinases are members of the serine-threoinine kinase with atypical placement of the catalytic lysine. The mutations of WNK cause pseudo-hypoalderonism type II (PHAII), a NaCl-senstitive hypertension (26). Subsequently, the WNKs are shown to regulate renal sodium transport through the SPAK and OSR1 kinases (27, 28). However, whether the WNKs and SPAK/OSR1 are associated with sex-dependent blood pressure regulation is still unclear.

In this study, we sought to understand if gender-associated differences in blood pressure are related to the regulatory mechanisms of renal sodium transporters NCC, NKCC2, and ENaC, as well as the upstream WNK and SPAK/OSR1 kinases in mice.

## Materials and Methods

### Blood pressure

The blood pressure of mice was measured by BP-98A (Softron, Tokyo, Japan). Four-month-old C57BL/6 mice were adapted to tail-cuff inflation for 20 min/d for 3 days, and blood pressure was measured from the 4th to the seventh day (29). For each mouse, a minimum of 25 blood pressure values were recorded daily. The average of the 25 measurements was recorded as the blood pressure of each mouse.

### Plasma and urine sodium

We kept both male and female mice (4 months old) in specialized metabolic cages to collect their urine. Mice were divided into two groups, each group having 3 to 5 males and females. One group fed on a normal diet (0.49% NaCl) for 2 weeks, then switched to a high-Na^+^diet (4% NaCl) for two weeks. Diets were purchased from Harlan Teklad (Madison, WI, USA) (30). The other group was fed on a normal diet 2 weeks; after that, we got their blood from orbital veins when mice were anesthetized by avertin (Sigma T480402) via intraperitoneally injected at a dose of 100 mg/kg according to the body weight of the mice(7, 31). Plasma was isolated from blood samples by centrifugation at 3,000 revolutions per minute for five minutes. The sodium concentration of urine and plasma was measured by a flame spectrophotometer. 

### Castration operation

We divided the male littermates into two groups: a castration and a sham operation group (32). For the surgeries, mice were anesthetized by avertin. Mice in the castration group underwent testicular removal surgery, whereas mice in the sham group underwent an identical operation without actual testicular removal. After the surgery, all the mice had a one-month recovery before we performed the following experiments: blood pressure measurement and urine sodium level evaluation. The laboratory animal center of Zhejiang Province approved all the experiments.

### Immunofluorescence and western blot

For immunofluorescence studies, the kidney was perfused with 20 ml of 4% paraformaldehyde after a flush with 15 ml of PBS, conducted via cardiac puncture (33). The kidneys were excised and dehydrated by soaking in 30% sucrose-PBS solution for 12 hr at 4 °C and embedded in Tissue-Tek OCT (Sakura Finetck). The 4μm-thick sections were stained with the primary antibodies: anti-ENaC antibody, followed by secondary antibody Alexa Fluor 555-Lebeled Donkey Anti-Rabbit IgG (red) and FITC Goat Anti-Rabbit (green). Fluorescent images were captured using a fluorescence microscope. For the western blot, the homogenate of kidneys was separated by polyacrylamide electrophoresis and then transferred to the hybridization membrane (blot) (7). The target protein is detected explicitly by the antibodies: anti-NCC and anti-NKCC2 (Stress Marq Biosciences Inc); anti-ENaC (Alomone Labs); anti-SPAK and anti-OSR1 (Cell Signaling); anti-WNK1 and anti-WNK4 (Alpha Diagnostic International).

### Statistical analysis

We used the two-tailed unpaired Student’s t-test to analyze the differences between male and female mice, castrated male and normal male mice. *P*<0.05 was deemed to be statistically significant.

## Results

### Blood pressure measurement and natriuresis

**Figure 1:**
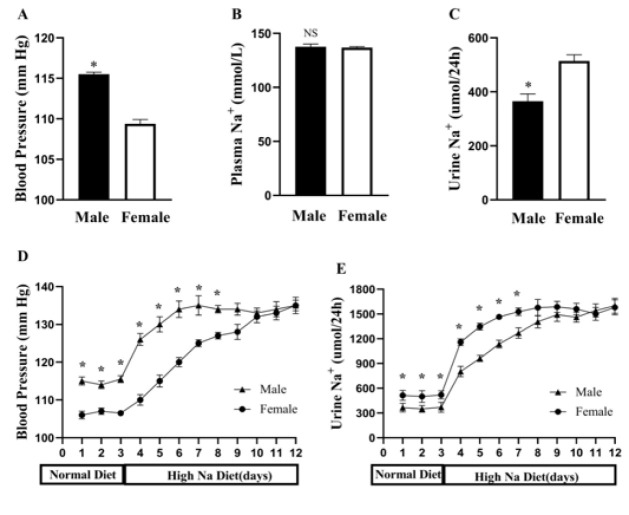
Blood pressure (A), plasma (B), and urine Na^+^ (C) on normal diet. Blood pressure (D) and urinary Na^+^ excretion (E) in male and female mice when switched from normal sodium (0.49% NaCl; day 1 to 3) to high sodium (4.9% NaCl; day 4–12) diet

On a normal diet, the blood pressure of male mice was higher than female littermates (Figure 1A). Although sodium concentration in plasma and urine showed no distinct difference between the genders (Figure 1B), 24 hr urinary Na^+^ excretion was significant lower in male than female mice (Figure 1C). To explore whether urinary Na^+^ excretion was related to sexual dimorphism of blood pressure, male and female mice were initially fed on normal diet following high-Na^+^ diet (2 weeks) in metabolic cages, urinary Na^+^ excretion and blood pressure was monitored daily. The difference between blood pressure (Figure 1D) and sodium excretion (Figure 1E) was widened (days 4–7) when switched to a high-Na^+^ diet. After day 8, there was practically no statistical difference in blood pressure and Na+ excretion between male and female mice. 

### Renal sodium transporter expression

**Figure 2:**
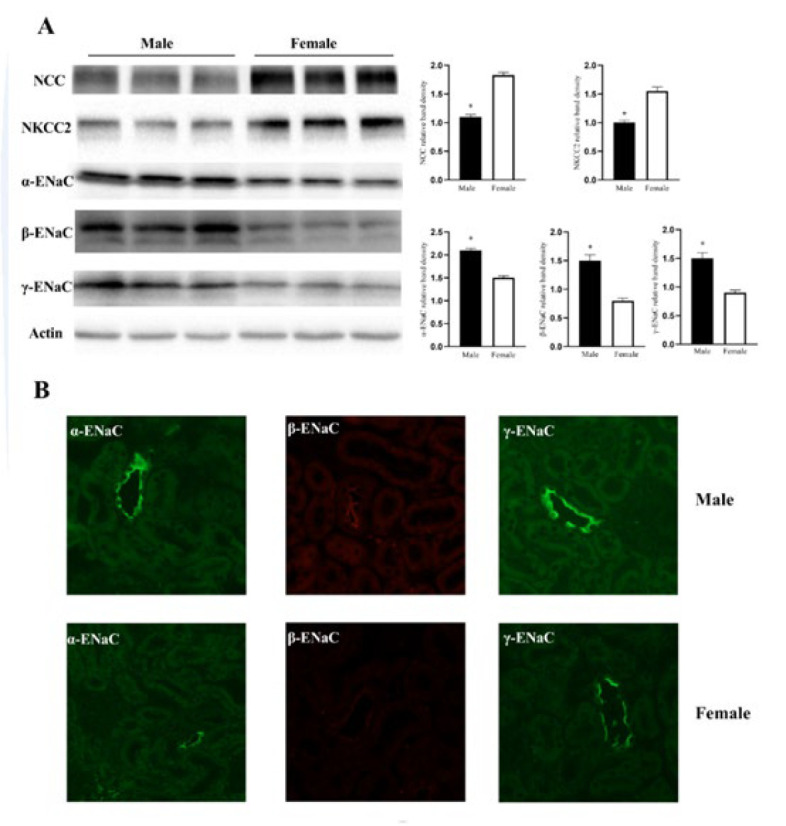
Expression level of NCC, NKCC2, α-ENaC, β-ENaC, and γ-ENaC in male and female mice

We next assessed the abundance of renal sodium transporters NCC, NKCC2, and ENaC (α, β, and γ) in the kidneys by western blot and immunofluorescent analyses. Figure 2 shows that α-ENaC, β-ENaC, and γ-ENaC protein levels were significantly elevated in male mice. 

### WNK4, SPAK and OSR1 abundance

**Figure 3:**
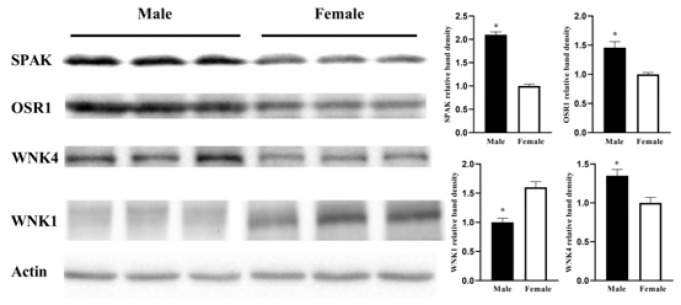
Abundance of SPAK, OSR1, WNK4, and WNK1 in male and female mice

WNK kinases, which lie upstream of SPAK and OSR1, are important regulators of renal sodium transporters. To investigate whether WNK1 and WNK4, along with downstream kinases SPAK and OSR1, are associated with gender-dependent blood pressure control, the western blot analysis was performed to evaluate the abundance of WNK1 WNK4 SPAK and OSR1. Figure 3 shows that, compared with female littermates, male mice exhibited a markedly increased abundance of WNK4, SPAK, and OSR1, while an apparent attenuation in the WNK1 abundance was observed.

### Blood pressure and natriuresis of castrated male mice

**Figure 4:**
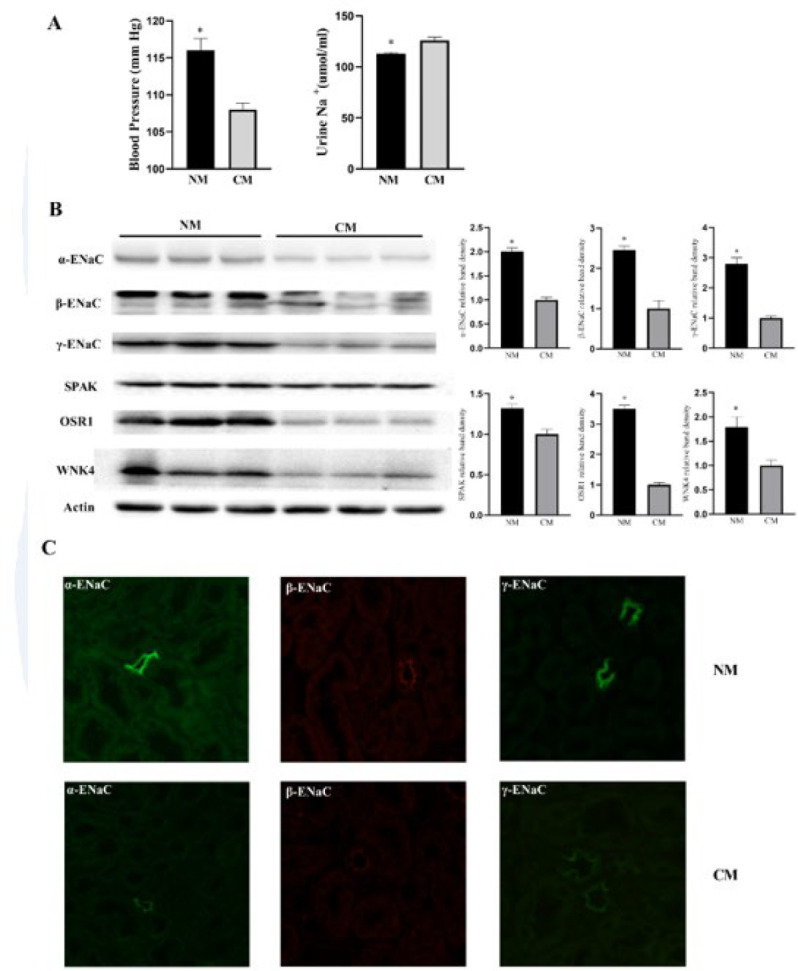
(A) Blood pressure and urinary Na^+^ excretion in uncastrated normal male mice (NM) and castrated male mice (CM). Western blot (B) and Immunofluorescent staining (C) analysis of the expression level of SPAK, OSR1, WNK4, α-ENaC, β-ENaC, and γ-ENaC in and CM

To further investigate the relationship between renal sodium reabsorption and gender-based differences in blood pressure, we executed the castration operation in male mice. The results showed that castrated males (CM) exhibited lower blood pressure and increased natriuresis compared with normal male (NM) littermates (Figure 4A). Besides, western blot and immunofluorescent assays revealed that the expression of α-ENaC, β-ENaC and γ-ENaC together with their upstream kinases WNK4 SPAK and OSR1 were reduced in CM mice (Figure 4B and Figure 4C).

## Discussion

Our research was designed to investigate the potential mechanisms of renal sodium transporters in regulating sex-dependent blood pressure. The rise of blood pressure in male mice was more dramatic than in females, which aligned with the slower increase in urinary sodium excretion when switched from a normal diet to a high-Na^+^ diet (days 4–7). These findings indicated that the differences in blood pressure between male and female mice are associated with variations in renal sodium reabsorption. In addition, we found that the expression of renal sodium transporters ENaC (α, β, and γ) and their upstream regulators SPAK, OSR1, and WNK4 kinases were higher in males than females. Furthermore, castrated male mice demonstrated characteristics similar to normal female mice, displaying lower blood pressure and increased natriuresis. These aligned with the observed decrease in the expression of renal sodium transporters ENaC (α, β, and γ) and upstream kinases WNK4 SPAK and OSR1. All the results suggested that ENaC and the upstream kinases participate in the gender differences in blood pressure regulation.

Previous studies have shown that decreased NCC and NKCC2 abundance can lead to Na^+^ wasting and hypotension (34-36). However, our data showed diminished expression levels of NCC and NKCC2 in male mice. This reduction in NCC and NKCC2 is inconsistent with the Na^+^ retention and increased blood pressure observed in males. It is hypothesized that the diminishment in NCC and NKCC2 may serve as a compensatory response to renal salt retention in male mice.

ENaC comprises α, β, and γ subunits and is crucial to renal Na^+^ reabsorption and blood pressure control. Numerous studies have highlighted that ENaC is subjected to sexual dimorphic regulation. An early study has shown that androgen enhances the expression of α-ENaC mRNA in rat kidneys (20). Han *et al.* later reported that estradiol injection induces the down-regulation of α- and γ-subunits of the ENaC in the ovariectomized rats (21). Our study elucidated a sex difference in ENaC with increased abundance in male mice. Building on former studies, the increase in ENaC expression has been linked to hypertension (37). Thus, the increased expression of ENaC may partially contribute to renal Na^+^ retention and higher blood pressure in male mice.

SPAK and OSR1, as members of the Ste20-related kinases family, are involved in regulating sodium homeostasis. Recent findings demonstrated that SPAK and OSR1 function as positive regulators in the modulation of NCC and NKCC2 (22). Therefore, the augmented expression of NCC and NKCC2 appears contradictory to the reduction in SPAK and OSR1 in female mice. Similar results have been reported that while the activity of SPAK and the abundance of ENaC are decreased, NCC’s expression will elevate (33). These illustrated the opposite alteration between NCC and SPAK. Further research has evidenced that SPAK modulates epithelial transport processes by up-regulating ENaC (38). Moreover, SPAK/OSR1 double knockout mice portray a reduced expression of ENaC compared to SPAK knockout mice (24), suggesting that OSR1 also mediates the regulation of ENaC. Collectively, these studies endorse the perception that SPAK and OSR1 may positively impact ENaC. Our results correspond with this conclusion, implying that the increased SPAK and OSR1 may account for the enhanced ENaC in male mice.

Results of our study also further elucidate the role of the WNK4-SPAK/OSR1 signal pathway in regulating renal sodium transport and blood pressure. Rafiqi *et al.* showed that the expressions of SPAK and OSR1 are up-regulated in mice with an increased WNK4 abundance (39). The WNK4 knockout mice model constructed by Shinichi Uchida *et al.* manifested a decrease in SPAK (40). These compelling results show that WNK4 is a positive regulator of SPAK and OSR1. The phenomenon of increased levels of kinases SPAK, OSR1, and WNK4 that we observed in male mice also supports this point. Previous studies affirmed that the hypertension in WNK4 mutant mice resulted from increased WNK4 activity (41, 42). Interestingly, the epidemiological data indicated that high blood pressure caused by WNK4 mutation is delayed in females compared to males (43). Additional reports elucidated that estrogen is inhibitory in the mRNA expression level of WNK4 (44). Thus, these results imply that WNK4 assumes a crucial function in sex-dependent blood pressure regulation and that increased WNK4 up-regulates SPAK and OSR1 in male mice. Considering the effect of SPAK and OSR1 on the activity of ENaC, we postulate that WNK4-SPAK/OSR1-ENaC signaling pathway may mediate one possible mechanism underlying the gender discrepancies observed in blood pressure.

The asterisk indicates *P*<0.05, NS means *P*>0.05, male versus female

(A) Western blot, bar graph shows density relative to female; (B) Immunofluorescent staining of α-ENaC, β-ENaC, and γ-ENaC in male and female mice. The asterisk indicates *P*<0.05, male versus female mice.

The right bar graph shows density relative to actin. The asterisk indicates *P*<0.05, male versus female mice.

The asterisk indicates means *P*<0.05, NM versus CM.

## Conclusion

Our study provides compelling evidence that ENaC functions as a significant determinant of sex-dependent differences in blood pressure, suggesting a possible involvement of the WNK4-SPAK/OSR1-ENaC signaling cascade. 

## Declaration

We have not used any AI tools or technologies to prepare this manuscript.
